# The immune checkpoint VISTA is associated with prognosis in patients with malignant uveal melanoma

**DOI:** 10.3389/fimmu.2023.1225140

**Published:** 2023-08-18

**Authors:** Nour el Imane Issam Salah, Farida Marnissi, Abdelhakim Lakhdar, Mehdi Karkouri, Mohamed ElBelhadji, Abdallah Badou

**Affiliations:** ^1^ Laboratory of Research on Neurologic, Neurosensorial Diseases and Handicap, Faculty of Medicine and Pharmacy, Hassan II University, Casablanca, Morocco; ^2^ Immuno-Genetics and Human Pathology Laboratory, Faculty of Medicine and Pharmacy, Hassan II University, Casablanca, Morocco; ^3^ Department of Pathological Anatomy, University Hospital Center (CHU) Ibn Rochd and Faculty of Medicine and Pharmacy of Casablanca, Hassan II University, Casablanca, Morocco; ^4^ Department of Adults Ophthalmology, 20 August Hospital 1953, CHU Ibn Rochd, Casablanca, Morocco; ^5^ Mohammed VI Center for Research & Innovation, Rabat, Morocco, Mohammed VI University of Sciences and Health, Casablanca, Morocco

**Keywords:** VISTA, PD1, CTLA-4, uveal melanoma, cancer immunotherapy, immune checkpoint inhibitor, immune microenvironment, prognostic factor

## Abstract

**Introduction:**

Uveal melanoma (UM) is a rare yet deadly tumor. It is known for its high metastatic potential, which makes it one of the most aggressive and lethal cancers. Recently, immune checkpoints such as Programmed cell Death protein-1 (PD1) and Cytotoxic T-Lymphocyte-Associated significantly increasing patient survival in multiple human cancers, especially cutaneous melanoma. However, patients with UMs were excluded from these studies because of their molecular characteristics, which tend to be widely different from those of cutaneous melanoma. This study aimed to analyze the expression of V domain Ig Suppressor T-cell Activation (VISTA), a novel immune checkpoint, to evaluate its prognosis significance and its correlation with PD1 and CTLA-4.

**Methods:**

Evaluation of VISTA, CTLA-4, and PD1 expression was performed through TCGA database analysis and immunohistochemistry using two independent cohorts with primary malignant UM.

**Results and discussion:**

Our results showed that VISTA expression was associated with tumor aggressiveness, T cell exhaustion, and the shortest median overall survival among patients. Surprisingly, PD1 protein expression was negative in all patients, whereas CTLA-4 expression was high in patients with advanced stages. Our findings suggest that VISTA may be a prognostic marker and an attractive treatment strategy for immunotherapy in patients with UM. Exploring its expression profile may predict response to immunotherapy and may lead to the improvement of precision therapy in malignant uveal melanoma patients.

## 1 Introduction

Uveal melanoma (UM) is the most common primary cancer of the adult eye (1). Owing to its rarity and complexity, it is one of the most challenging and hardest cancers to study. Its aggressiveness, invasion potential, and high metastasis susceptibility in almost half of the patients impact its prognostic value and eventually decrease patient survival (1, 2). Various clinical and histological aspects are related to worse prognosis in patients with UM, including tumor location, tumor thickness, large tumor basal diameter, involvement of the ciliary body, epithelioid subtype, and cytogenetic features (3). Furthermore, when small, UM cells can be killed by the immune system. However, in later stages, immune checkpoints help the tumor grow and spread by weakening the immune system (4). To identify and eliminate these cancer cells, some immune checkpoints are turned on or overexpressed to stop the immune response against the tumor (5). UM may use these pathways to avoid being attacked by the immune cells. This may lead to tumor escape from the immune system, resulting in tumor growth and spread (6).

While Programmed cell Death protein-1 (PD1), Programmed Death Ligand-1 (PD-L1), and Cytotoxic T-Lymphocyte-Associated protein-4 (CTLA-4) blockade showed successful responses in patients with cutaneous melanoma (7, 8), patients with UMs were excluded from these studies because their molecular characteristics tend to be widely different from cutaneous melanoma (9, 10). Nevertheless, even with the discovery of different therapies, no definitive cure has been established, especially in patients with metastatic UM (11). Although surgery and radiotherapy are conservative treatment options for a subset of patients, up to one-third of UM may ultimately metastasize (12). This lack of effectiveness may be due to the exceptional microenvironment of the eye and the special mechanisms by which UM escapes the immune response. In fact, little is known about the implication of these mechanisms in this type of cancer, and the cause of the limited response of UM patients to immunotherapy is still unclear and blurred. Hence, our current challenge remains in the identification of additional suppressive pathways.

VISTA (V-domain Ig Suppressor T-cell Activation) encoding C10orf54, also known as V-set immunoregulatory receptor (Vsir), is a type I transmembrane protein located on chromosome 10q22.1. Demonstrated to dull T cell activation, VISTA is highly expressed in myeloid cells (such as macrophages, monocytes, and dendritic cells), on T cells (such as CD4+ and CD8+), and negatively expressed on B cells in several *in vitro* and *in vivo* studies (13). VISTA is a novel immune checkpoint that regulates T-cell function. Its role has been studied in several types of cancer, including gastric and ovarian cancers (14, 15). In murine melanoma tumor models, VISTA mAb treatment induced the activation of T cells, suppression of tumor growth, and stimulation of the immune response (16); proving its role in regulating the tumor immune response.

Little is known about the expression of distinct immune checkpoints and their function in UM, and no data exists regarding VISTA expression or its impact on the UM microenvironment. In the present study, we aimed to analyze VISTA expression within the tumor microenvironment of UM patients, to examine its association with clinicopathological features, to evaluate its prognostic factor, and to correlate it with PD1 and CTLA-4, two immune checkpoints already studied in UM pathology. Here, we propose that VISTA may be a novel engaging immune checkpoint and a new target for cancer immunotherapy in UM patients.

## 2 Materials and methods

### 2.1 Validation step

**Figure 1:**
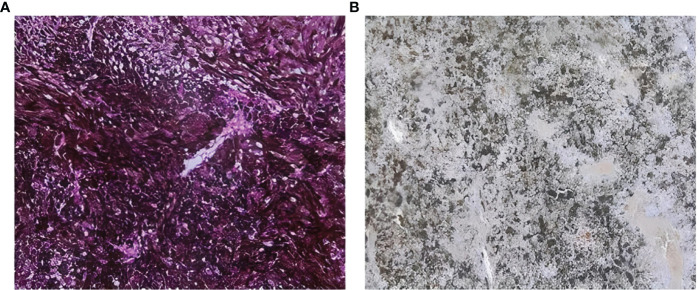
Sections counterstained with Giemsa coloration **(A)** section showing high pigmentation with melanin shown as black or brown color **(B)** section with Giemsa counterstaining showing that melanin brown pigment is transformed successfully to a green color.

One of the most challenging characteristics while validating an immunohistochemical procedure in melanocytic diseases -such as UM- is the melanin pigment. Melanin is a pigment that appears granular brownish to black, making the revelation of immunoreactivity with diaminobenzidine (DAB), which has the same color, impossible. Thus, immunostaining of tumor and immune cells is obscured by melanin. Since most of our sections were highly pigmented, we tended to use Giemsa counterstaining. Therefore, separate formalin-fixed paraffin-embedded (FFPE) sections of UMs tissues were used for KI67 antibody immunohistochemistry evaluation to test the utility of this protocol. This allowed us to apply the same protocol to the investigation of other antibodies that are only meant for research use. Giemsa counterstaining is an effective and inexpensive alternative to other bleaching methods. Therefore, to facilitate immunohistochemical examination and analysis, the melanin brown pigment was successfully transformed into a green color in all cases (**Figure 1**).

### 2.2 Patients and specimens

A total of 105 primary malignant UM patients were included in this study: 25 Moroccan UM patients who underwent enucleation as well as 80 American UM patients from The Cancer Genome Atlas (TCGA) dataset. Given the rarity of this type of tumor, fresh tissue samples were difficult to obtain to perform further experiments. Under these circumstances, FPPE tissues were collected through a multi-center study. Nevertheless, mRNA expression data were selected from the cBioPortal for cancer genomics (https://www.cbioportal.org/).

The median was used as a cut-off for lower and higher gene expression to cluster different groups. An operating sheet was created with major clinicopathological parameters susceptible to having a prognostic role in this type of cancer in both cohorts.

### 2.3 Histopathology and immunohistochemistry

Eventually, 3–4 μm sections were sliced from FFPE blocks that were wisely chosen and rated by expert pathologists, whereas inadequate blocks were excluded. To ensure the presence of viable tumor cells, Hematoxylin and Eosin slides were prepared, examined, and reviewed to determine pigmentation, necrosis, tumor thickness, stages, and histological subtypes. Patients were classified according to their histopathological subtypes as epithelioid cells, spindle cells, or mixed cells if more than 10% of the tumor showed both spindle and epithelioid cells. Therefore, patients with diagnoses other than UM, without histological confirmation, and/or non-exploitable blocs were excluded.

The technique was performed manually according to our laboratory protocol, which was edited to suit our pigmented UM tissues. Briefly, each section was first subjected to heat (75°C for 1 h and 45°C overnight), followed by demasking of antigenic sites at pH9 using PT Link, followed by Giemsa 10% counterstaining. After peroxidase, a series of incubations with primary antibodies were executed: anti-VISTA (Monoclonal Mouse; 1:50; Clone UMAB271; OriGene Technologies), anti-PD1 (Monoclonal Mouse; clone DBM15.5, ready to use), or anti-CTLA-4 (Monoclonal Mouse; 1:250; Santa Cruz). Human tonsil, lung squamous carcinoma, and human appendix tissues were used as controls for VISTA, PD1, and CTLA-4 expression, respectively. Likewise, for every tissue, an isotype control IgG1 (LifeSpan BioSciences; 1:200; LS-C355904; MOPC-21) was used, followed by HRP EnVision FLEX. Next, antibody staining was visualized using DAB as a chromogen. Finally, hematoxylin counterstaining was used to obtain better visualization of tissue morphology.

### 2.4 Immunohistochemical evaluation

Immunostaining was performed by two independent senior expert pathologists. *Eyeballing* counting method with an eyepiece grid was assessed using microscope. In the case of homogeneous labeling, the assessment was made on the whole section. In the case of heterogeneous labeling, the assessment was carried out on a minimum of three randomly selected fields in the areas of interest with low magnification, taking into account cell density and considering the fact that a x400 field of a moderately cellular tumor contains about 500 to 700 tumor cells.

Besides, we considered both the percentage of stained cells and the intensity of immune and tumor cells. The overall immunoreactivity score, ranging from 0 to 300, was determined by considering the percentage of immunostaining (scaled from 0 to 100%) multiplied by the dominant intensity pattern (scaled from 0 to 3). Immunostaining was detected in all sections under a light microscope for analysis and scoring. VISTA, PD1, or CTLA-4 expression was indicated by brown staining, which was considered positive if any cell had undergone membranous and/or cytoplasmic staining. Scoring is based on the proportion of positive infiltrating tumor/immune cells relative to all tumor/immune cells (positive and negative), with a membranous and/or cytoplasmic staining. The result is expressed as a percentage of cells rounded to the nearest five, taking into account all marked intensities. When it comes to the background signals, all blurred areas were excluded from the counting.

### 2.5 Statistical analysis

Data were descriptively analyzed to evaluate statistical frequencies, which were executed using the Statistical Package for the Social Sciences (SPSS) version 25 (IBM SPSS, SPSS Inc., Chicago, IL, USA). Statistical analysis and graphs were generated using GraphPad Prism 6.0 software (GraphPad Software, Inc., La Jolla, CA, USA). RNA-seq data were visualized as log 2 of RSEM (TPM). DESeq2 normalization was performed for each sample to obtain one scaling factor per sample to compare the mRNA expression profiles of these genes in the UM microenvironment. The database was log-transformed for further analyses.

Non-parametric Mann–Whitney tests were conducted to compare low and high expression of the different clusters, and P values were considered significant when P<0.05. Spearman’s rank correlation was performed to evaluate the correlation functions. Kaplan–Meier survival was used to analyze the overall survival rate of the patients.

### 2.6 Ethical approval

The study was conducted in accordance with the institutional guidelines. Ethical approval for our protocol was obtained from the Institutional Ethics Committee of Biomedical Research of Casablanca (N°04/21). All the included patients provided oral and written informed consent.

## 3 Results

### 3.1 Patient characteristics

Clinical and pathological features were included in all patients. A total of 80 patients were investigated in TCGA database; 56% (45/80) were males and 43% (35/80) were females. Elderly patients were found in 55% (44/80) of the cases, with extreme ages ranging from 35 to 86 years old. Cases were divided into the spindle subtype (37,5%), mixed subtype (46,3%), and epithelioid subtype (16,3%). Therefore, 36 low-stage and 44 high-risk patients were included in the study. The second cohort with 25 Moroccan patients, was characterized by 56% females and 44% males. Most of our patients were aged < 60 years, with ages ranging between 27 and 79 years. In addition, 40% (10/25) of UM patients with low-stage were included versus 60% (15/25) with high-stage UM (**Table 1**).

**Table 1:** Clinical features in a set of 2 cohorts with uveal melanoma, both number of patients and percentage are presented.

	TCGA database Clinicopathological parameters (N=80)		Moroccan cohort Clinicopathological parameters (N=25)	
	N	%	N	%
Gender				
Female Male	35 45	43,8 56,3	*14* *11*	*56* *44*
Age				
< 60 years ≥ 60 years	36 44	45 55	19 6	76 24
Histological subtype				
Spindle cell Mixed cells Epithelioid cell	30 37 13	37,5 46 16,3	10 7 8	40 28 32
Stage				
Low stage High stage	36 44	45 55	10 15	40 60

### 3.2 Immune checkpoints expression pattern revealed highest expression of VISTA in UM microenvironment

**Figure 2:**
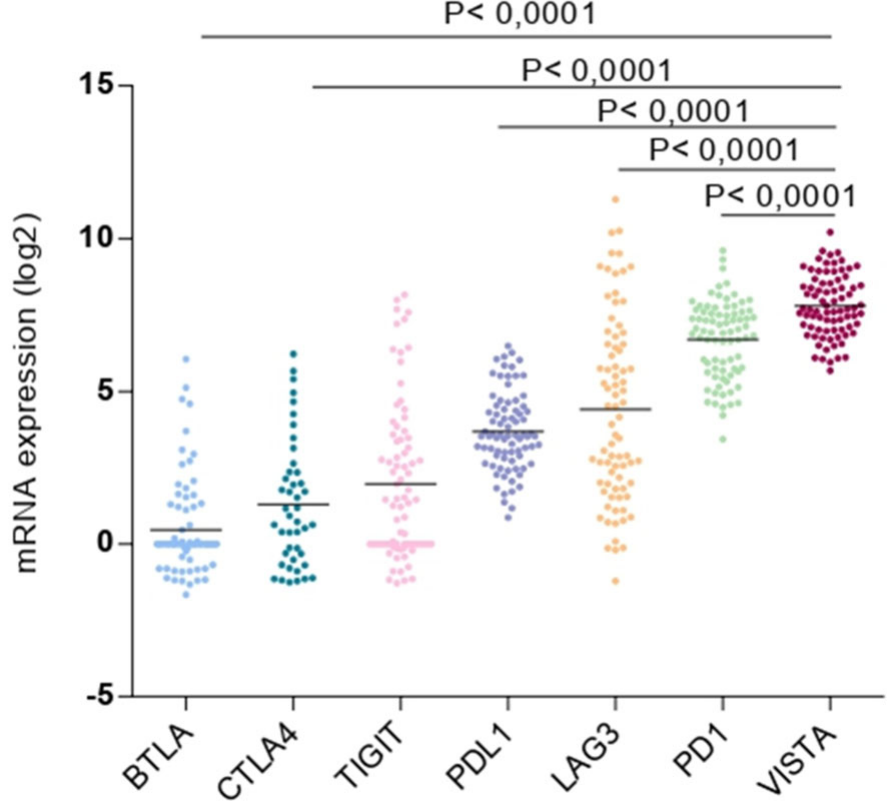
Expression of multiple immune checkpoints (BTLA, CTLA4, TIGIT, PD-L1, LAG3, PD1 and VISTA) in uveal melanoma microenvironment.

Here, we explored the expression variety of multiple immune checkpoints that are present in our rare tumor database of TCGA. Consequently, among the mRNA expression genes encoding for immune checkpoints, for instance, B and T Lymphocyte Associated (BTLA), CTLA4, T Cell Immunoreceptor with Ig and ITIM Domains (TIGIT), PD1, PD-L1, lymphocyte-activation gene-3 (LAG3), and VISTA. The latter was found to be the most overexpressed gene in the UM microenvironment (**Figure 2**).

### 3.3 VISTA mRNA expression is associated with the most aggressive clinicopathological features in UM patients

**Figure 3:**
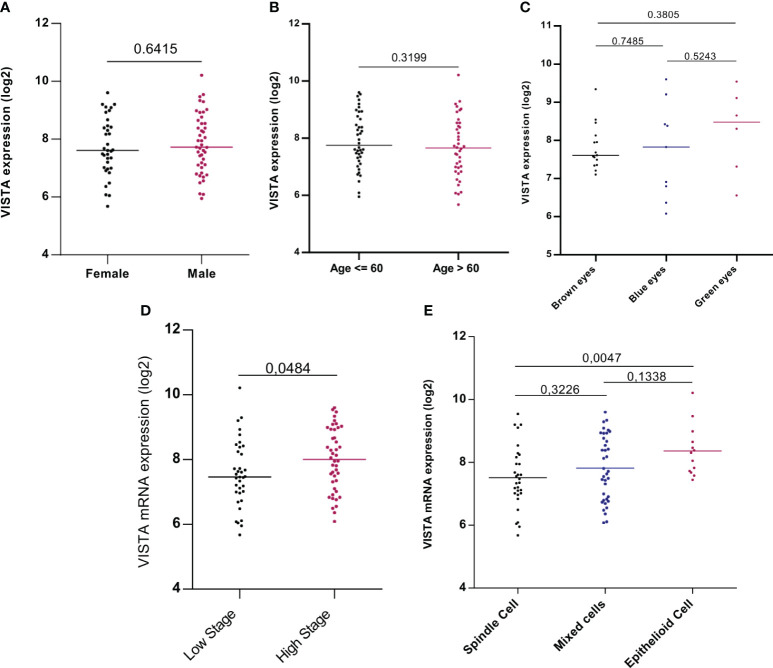
VISTA mRNA expression and its association with clinicopathological parameters: gender, age, eye color, stage, and histological subtypes of UM patients in the TCGA database **(A)** Expression of VISTA gene showed no difference between males and females (P=0,6415) **(B)** VISTA mRNA expression was not influenced by age of uveal melanoma patients (P= 0,5629) **(C)** VISTA gene level expression comparison between eye colors **(D)** VISTA mRNA expression was strongly expressed in patients with high stage uveal melanoma compared to those with low stage (P=0.0484) **(E)** Comparison of VISTA gene expression between different cell types of uveal melanoma, significance was noticed between spindle and epithelioid cell type that is marked with strong expression of VISTA (P=0.0047).

The association between VISTA and clinicopathological parameters was investigated in order to assess its prognostic factor in patients with UM. TCGA analysis revealed that pathological TNM stages (P=0.0484) and histological subtypes, especially between spindle and epithelioid cells (P=0.0047), were significantly associated with worse patient prognosis. However, no statistically significant correlations were found with other clinicopathological parameters such as sex (P=0,6415), age (P= 0,5629), or eye color (**Figure 3**).

**Figure 4:**
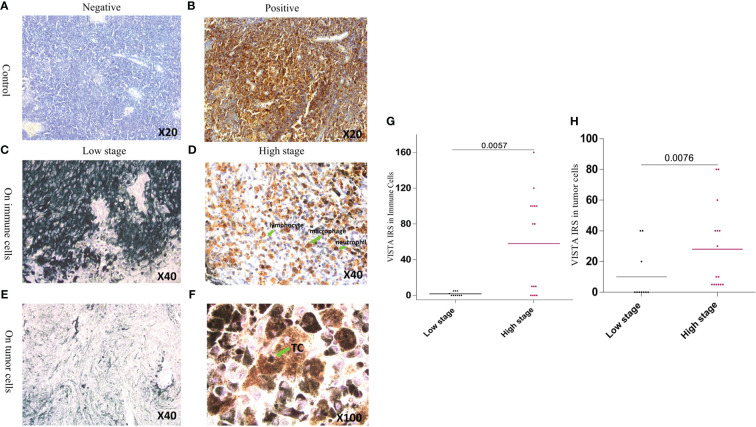
Expression of VISTA by immunohistochemistry in both controls and UM tissue samples **(A)** Negative staining of human tonsil with IgG-1 negative control **(B)** Human tonsil with positive VISTA immuno-staining **(C)** VISTA negative expression in immune cells in low stage patients with uveal melanoma **(D)** Immune cell positive immunostaining of VISTA expression in high stage uveal melanoma patients **(E)** Tumor cell negative immunostaining of VISTA expression in low stage uveal melanoma patients **(F)** Tumor cell positive immunostaining of VISTA expression in high stage uveal melanoma patients **(G)** Comparison of the association of VISTA immunoreactivity between low and high stage in immune cells (P=0.0057) **(H)** Comparison of the association of VISTA immunoreactivity between low and high stage in uveal melanoma cells (P=0.0076).

To further confirm our results, protein analysis was performed using immunohistochemistry to quantify VISTA,PD1, and CTLA-4 expression. First, the clinicopathological parameters of our Moroccan cohort were explored to analyze their correlation with VISTA protein. VISTA staining was found to be expressed both on tumor cells in 72% of cases and on immune cells in 76% of cases, which was noted essentially in lymphocytes, monocytes, macrophages, and neutrophils with membranous and/or cytoplasmic immunostaining. Specifically, overall percent VISTA protein expression in the membrane, cytoplasm or both is indicated in **Table 2**. VISTA protein was also expressed in endothelial cells in 16% of the cases. In particular, VISTA immunostaining has been observed to have focal positivity in a few cases. The human tonsil was used as a positive control for VISTA antibody and showed intense positive staining (**Figure 4B**), whereas the IgG1 antibody was negative (**Figure 4A**).

**Table 2:** Overall percent of VISTA protein expression in the membrane and/or cytoplasm of immune and tumor cells of uveal melanoma sections.

Staining	Membranous		Membranous and cytoplasmic		Cytoplasmic	
	N	%	N	%	N	%
**VISTA protein expression in immune cells (N=19)**	7	37	7	37	5	26
**VISTA protein expression in tumor cells (N=18)**	4	22	6	33	8	45

Generally, VISTA expression is positive in epithelioid cells, which correlates with a poor prognosis. In the lower stages, it was rarely expressed; therefore, none of the patients with a high stage of malignant UM showed negative staining. Eventually, all patients with the worst aggressive stage expressed VISTA protein. Higher stages displayed high VISTA protein expression, in contrast to lower stages in both immune cells (P= 0,0057) and UM cells (P= 0,0076) (**Figure 4**).

### 3.4 VISTA correlation with PD1 and CTLA-4

**Figure 5:**
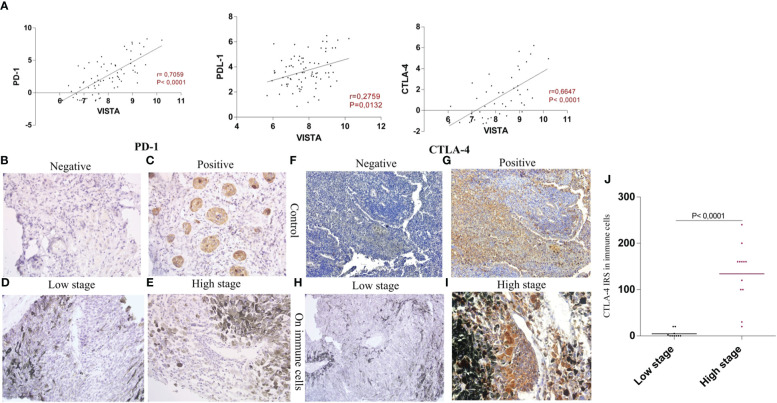
Evaluation of VISTA, PD1 and CTLA-4 expression within UM patients **(A)** VISTA mRNA expression and its correlation with PD1 (P<0.0001, r=0.7059), PD-L1 (P=0.0132, r=0.2759), and CTLA-4 (p<0.0001, r= 0.6647) **(B)** IgG1 isotype control shows negative expression of PD1 in lung squamous carcinoma **(C)** Positive expression of PD1 in carcinoma cells used as a control **(D)** Negative staining of PD1 in low stage uveal melanoma patients **(E)** Negative staining of PD1 in high stage uveal melanoma patients **(F)** IgG1 negative expression of CTLA-4 in the human appendix tissue **(G)** CTLA-4 positively stained immune cells in the human appendix tissue section **(H)** Negative staining of CTLA-4 protein in low stage uveal melanoma patients **(I)** Positive staining of CTLA-4 in high stage uveal melanoma patients observed exclusively in immune cells **(J)** comparison of CTLA-4 protein expression in low stage versus high stage uveal melanoma patients.

High correlations were found between VISTA, PD1 (P<0.0001, r=0.7059), and CTLA-4 (p<0.0001, r= 0.6647), compared to PD-L1 (P=0.0132, r=0.2759), which showed the lowest correlation. Therefore, both VISTA and CTLA-4 were highly expressed at higher stages, confirming a positive correlation (**Figure 5A**).

In our sections, all slides lacked PD1 immunostaining in either immune and tumor cells (**Figures 5D, E**). However, lung squamous carcinoma, which is a PD1 control, showed positive staining in 100% of carcinoma cells (**Figure 5C**), in comparison with IgG1 isotype control that showed negative staining (**Figure 5B**), allowing us to confirm sample antigenicity. In the human appendix tissue sections, all cells were negative when stained with IgG1 isotype control (**Figure 5F**), however, high positive staining with CTLA-4 atibody was noticed as shown in **Figure 5G**. In addition, CTLA-4 displayed membranous and/or cytoplasmic positive staining on immune cells, especially in tumor infiltrating lymphocytes (TILs) (**Figure 5I**). Accordingly, positive CTLA-4 protein staining was observed in 64% of the cases (**Figures 5I, J**); our analysis also revealed that strong CTLA-4 immunoreactivity was observed in higher stages compared to patients with lower stages (P<0,0001) (**Figure 5H**).

### 3.5 Evaluation of the prognostic significance of CD8+ T cells, CD4+ T cells, and regulatory T cells and their association with VISTA mRNA expression in UM microenvironment

**Figure 6:**
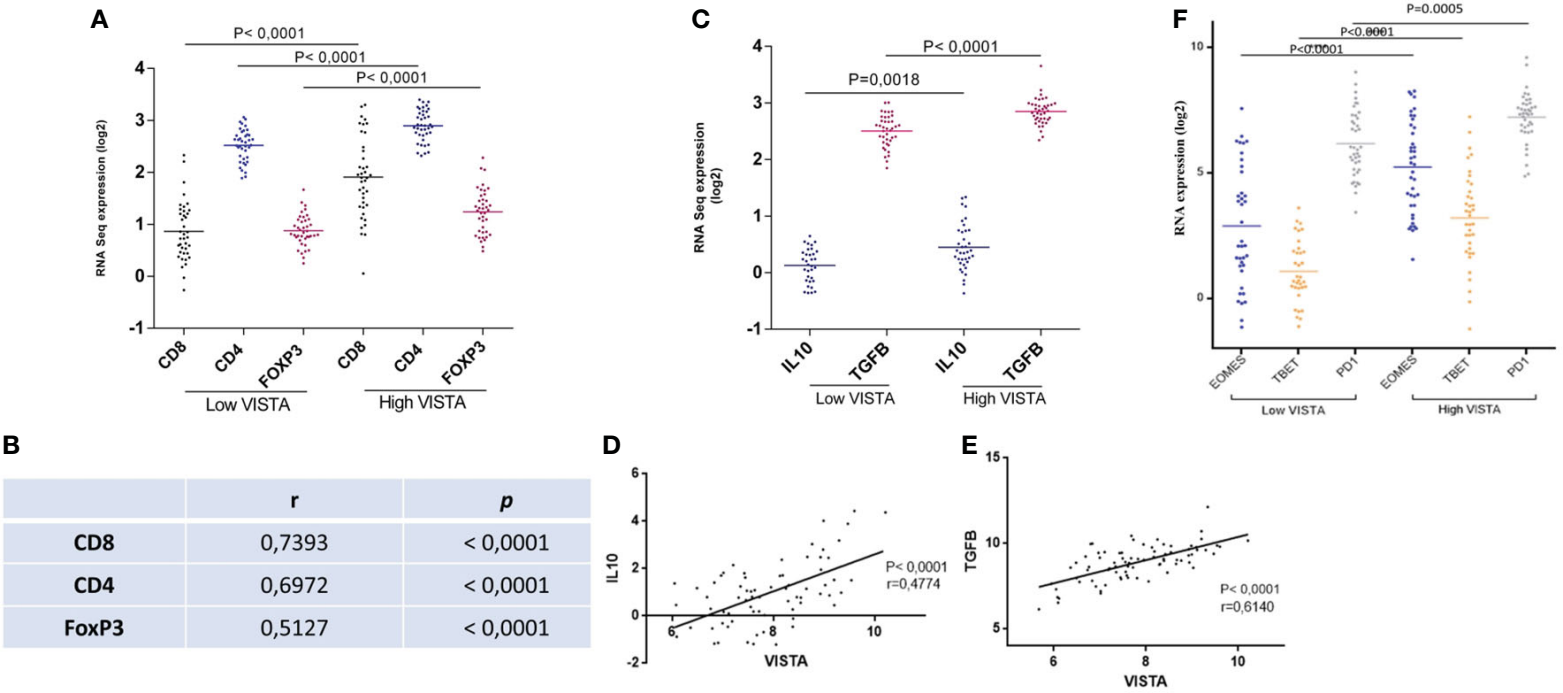
Low versus high VISTA expression is linked to T cell subtypes markers as well as anti-inflammatory cytokines in the TCGA database of UM patients **(A)** High infiltration of CD8, CD4, and FoxP3 is associated significantly with high VISTA mRNA expression **(B)** VISTA mRNA expression and its correlation with CD8, CD4, and FoxP3 (r=0.7393, P<0.0001; r=0.6972, P<0.0001; r=0.5127, P<0.0001) **(C)** High expression of VISTA is associated to high expression of IL-10 (P=0.0018), and TGFB (P<0.0001) **(D)** Positive correlation between VISTA and IL-10 (r=0.4774, P<0.0001) **(E)** Positive correlation between VISTA and TGFB (r=0.6140, P<0.0001) **(F)** Overexpression of VISTA is significantly related to T cell exhaustion markers (EOMES, TBET, and PD1) in the UM microenvironment of patients from the TCGA database (P<0.0001).

Since we already found using immunohistochemistry that VISTA is expressed on T cells; we attempted to gain insights into UM microenvironment and its molecular signature in the TCGA cohort. Therefore, association of VISTA expression levels and T cells markers for CD8+T cells, CD4 +T cells and regulatory T cells were assessed in order to evaluate their prognostic significance as well as their potential role in regulating anti-tumor immunity in UM microenvironment. When comparing low and high VISTA expression, a high infiltration of T cells markers genes is significantly noticed when VISTA is highly expressed (P<0.0001) (**Figure 6A**). Study of the correlation showed significantly positive correlation for CD8, CD4 and FoxP3 respectively (r=0.7393, P<0.0001; r=0.6972, P<0.0001; r=0.5127, P<0.0001) (**Figure 6B**).

The association between VISTA and anti-inflammatory cytokines, such as transforming growth factor beta (TGFβ) and interleukin 10 (IL-10), were also studied. They are secreted by FoxP3, and are known to inhibit other immune cell functions. Interestingly, they were found to be significantly related to high VISTA expression (**Figure 6C**). Moreover, a positive correlation between VISTA expression and IL-10/TGFB in UM microenvironment was noticed in **Figures 6D, E** respectively (r=0.4774, P<0.0001; r=0.6140, P<0.0001). This suggest that VISTA may contribute to an immunosuppressive microenvironment in the context of UM.

### 3.6 VISTA overexpression is associated with T cell exhaustion in UM microenvironment

To go even further in our hypothesis, the functional status of CD8 T cells was also assessed evaluating markers of T-cell exhaustion. The last is characterized by the presence of high EOMES, TBET, and PD1. Our results show that patients with high VISTA mRNA expression displayed high expression of EOMES, TBET and PD1 in comparison with low VISTA mRNA expression (P<0.0001) (**Figure 6F**).

### 3.7 Clinical significance and prognostic evaluation of VISTA in UM microenvironment

**Figure 7:**
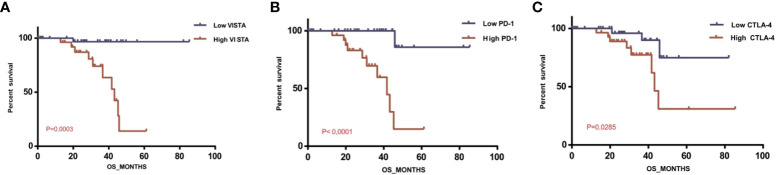
Overall survival of patients with uveal melanoma depending on VISTA, PD1 and CTLA-4 expression levels in the TCGA database **(A)** Overall survival rate depending on VISTA mRNA expression level (P=0.0003); **(B)** Overall survival rate depending on PD1 mRNA expression level (P<0.0001); **(C)** Overall survival rate depending on CTLA-4 mRNA expression level (P=0.0285).

To evaluate the prognostic significance of VISTA, UM patients of the TCGA database were divided into two clusters: lower VISTA mRNA expression and higher VISTA mRNA expression. In addition, we sought to elucidate the effect of PD1 and CTLA-4 mRNA expression on survival. We found that high CTLA-4, high PD1, and high VISTA levels decreased patient survival, with a significant overall survival (P=0.0285, P<0.0001, and P=0.0003, respectively). Unpredictably, we found a stable overall survival in patients with low VISTA expression, and the shortest median overall survival was observed in patients with high VISTA expression, proving its worse prognosis status (**Figure 7**).

## 4 Discussion

Over the past decades, therapeutic strategies have revolutionized cancer treatment (17). Still, no treatment has been totally effective for UM patients to date (11). Therefore, a better understanding of the molecular signatures of UM is necessary. To the best of our knowledge, this is the first study to assess the prognostic value of VISTA in UM patients.

The Cancer Genome Atlas (TCGA) is a database with a large amount of genomic data that enables us to study the immune microenvironment and molecular pathways involved in UM progression and invasion. It is also an interesting tool for confirming research studies worldwide because of its open accessibility. Here, we performed transcriptomic analysis as it is an advantageous tool filling the gap between genomics and proteomics and guiding translational and clinical studies (18, 19).

In this dataset, we analyzed the recently discovered immune checkpoints, VISTA, LAG-3, PD-L1, PD1, TIGIT, CTLA-4, and BTLA, to compare their expression within the UM immune microenvironment. Except for VISTA, these available genes in our TCGA database have been previously studied in UM (20–23). Remarkably, VISTA expression was the highest (**Figure 2**). RT-PCR analysis and flow cytometry of several mouse tissues determined that VISTA is not expressed in the normal eye. Besides, higher expression has been observed in the thymus, spleen, and bone marrow (13). This supports the fact that VISTA may be highly expressed in UM patients but not in healthy individuals, possibly playing a major role in cancer progression.

Clinicopathological features of our patients were assessed according to the AJCC classification which considers ciliary body involvement and episcleral extension. Subsequently, both cohorts showed no significant differences in sex or age (**Figures 3A, B**).

Indeed, no gender preference is stated for this disease, while others claim that UM is most common in males (24). It has also been found that UM is common in elderly patients and is linked to lower overall survival. In addition, fair skin, light eye color, and Caucasian populations are among the risk factors for UM (25). Consistent with our data reporting that VISTA is associated with clinicopathological features linked to poor prognosis in the UM microenvironment, for instance, advanced stages and epithelioid cells (**Figures 3D, E**); it was reported that the presence of epithelioid cells is associated with UM progression and metastasis development. However, better survival was assigned in patients with the spindle subtype (1).

Subsequently, immunohistochemistry was used to quantify VISTA protein expression and different scoring approaches have been assessed in this study in order to confirm transcriptomic results. Eventually, both the percentage of positive immunostaining and the intensity were considered. Previous studies have only limited the expression of VISTA to immune cells (16, 26). Conversely, recent studies have provided clear evidence that both immune and tumor cells express it (14, 27, 28). Remarkably, immune cells, tumor cells, and endothelial cells all were shown to express VISTA (**Table 2**), as found in patients with gastric cancer (14). Likewise, UM harbors higher expression of CD4+ CD8+ and CD11b+ cells, which are constitutively expressed by VISTA (29). Consistent with these results, other studies have reported that VISTA protein is expressed on T cells, and its high expression is associated with high levels of CD3+, CD4+, and CD8+ T cells (30).

In the same cohort, the results indicate that the VISTA protein has elevated expression in higher stages. This suggests that it would have a worse prognosis in UM patients with advanced stages. Indeed, it has been proven that VISTA has a worse prognosis in multiple types of cancers, for instance, ovarian cancer, human non-small cell lung cancer, cutaneous melanoma, glioma, and colon cancer (15, 27, 29, 31, 32). In contrast, VISTA has a good prognosis in malignant pleural mesothelioma and breast cancer, specifically in triple-negative patients, cervical cancer, and endometrial cancer, with better overall survival in patients with higher VISTA expression. Single-cell analysis and proteomic studies using both immunohistochemistry and quantitative immunofluorescence results reported lower VISTA expression levels in adjacent tissues than in breast cancer cells. Interestingly, VISTA expression was significant in terms of overall survival rate (33–36). In our sections, VISTA staining was detected mainly in T cells, monocytes, macrophages, and neutrophils. Consistent with our findings, VISTA expression in T cells and in the myeloid lineage leads to their regulation and was found to be associated with poor prognosis. Consequently, when VISTA is upregulated, the levels of IL10, IFN gamma, and FOXP3 decrease (27–29). The latter suggests the role of VISTA in regulating the tumoral immune response; its upregulation is therefore an independent marker of poor survival.

Since anti-PD1 (nivolumab, pembrolizumab) and anti-CTLA-4 (ipilimumab) have been approved by the U.S. Food and Drug Administration (FDA), and since VISTA was found to be correlated with these two immune checkpoint inhibitors (**Figure 5A**). Consistent with the findings of Böger et al., we decided to study their expression in patients with UM to further support our findings, since mRNA expression analysis solely cannot predict protein levels (14).

PD1, also known as CD279, is a transmembrane glycoprotein expressed in activated T and B lymphocytes, NK cells, and monocytes. It contains two tyrosine kinase domains in its cytoplasmic tail (37). Once activated by binding to either PD-L1 or PDL-2 ligands, the TCR and BCR signaling pathways are blocked (38).

CTLA-4, also known as CD152, is a membrane glycoprotein that binds to ligands of the B7 family (CD80 and CD86) on the surface of APCs, and is highly similar to CD28, which is a stimulatory checkpoint molecule. By binding to its ligand, CTLA-4 suppresses signaling pathways in T cells, leading to T cell anergy, fatigue, and a diminished T cell immunological response (39). CTLA-4 might suppress T-cell-mediated antitumor immune responses by attenuating tumor-specific T-cell activation before these T-cells eradicate the tumor. Its blockade was thought to increase T cell-mediated antitumor immunity by eliminating this inhibitory signal (40).

Numerous clinical trials have demonstrated the function of VISTA in the inhibition of T cell responses and its overexpression after anti-PD1 therapy. Otherwise, the response of melanoma patients to immunotherapy induces resistance to anti-PD1 and anti-CTLA-4 treatment (41, 42). Increased expression of VISTA was observed not only after treatment with anti-PD1 alone but also in combination with CTLA-4, proving the activation of VISTA pathway after these treatments and explaining their non-efficacy in various aggressive cancers (41).

PD1 protein expression was astonishingly not noticed in any of our samples. To emphasize, our UM sections did not show any expression of PD1 protein (**Figures 5D, E**). This suggests that VISTA and PD1 pathways are non-redundant in the cancer immune response, consistent with the results reported by Liu et al. (43). Similarly, it has been shown that UM undergoes decreased PD1 and PD-L1 protein expression levels in patients compared with other tumors such as cutaneous melanoma (44). In contrast, Jiang et al. observed PD1 expression in 50% of primary UM cases, and higher expression of PD1 in tumor cells was linked to progression of UM cells and lower patient survival (45). However, CTLA-4 protein was detected only in inflammatory cells, specifically in monocytes and T cells, which are mainly TILs, and was noticed in higher stages of UM (**Figure 5I**). Similar to anti-PD1 agents, treatment with anti-CTLA-4 antibodies showed discouraging results in patients with UM, with a minimal response rate that did not exceed 10% (46).

As the presence of PD1 is required for PD1/PD-L1 interaction, its expression was found to vary among studies. The prognostic value of the PD1/PD-L1 pathway as a regulator of T cell activation in UM was assessed. An *in vitro* study using RT-PCR and flow cytometry analysis revealed that PD-L1 was constitutively expressed in five of nine primary UM cell lines. However, immunohistochemical analysis showed that PD-L1 protein was not expressed in patients with primary UM and was negatively expressed in all cases. However, its expression has been observed in patients with metastatic UM (47). This may explain that PD-L1 expression was influenced by the eye microenvironment. Another study demonstrated that almost half of patients with primary UM express PD-L1 protein (48). The mRNA expression in the same study analyzing two different cohorts established that the first cohort had a favorable prognosis and decreased infiltration of TILs, whereas the second cohort showed no significance in terms of overall survival. Our data suggest that the expression of PD1 and PD-L1 may be specific to each tumor type; in particular, it may depend on the tumor’s molecular phenotype. Although PD1 and PD-L1 were found to be expressed in various studies, their expression was very low in UM compared to other types of cancers. This low expression may explain the non-effectiveness of anti-PD1 therapies tested to date in the treatment of patients with UM, suggesting that it may be less effective in treating this type of tumor.

The key question of this study is why the inflammatory cells present in our UM sections do not present any PD1 staining?

Positive significant correlation was found between the average mRNA and the average protein expression (49); however, we here found that PD1 protein is different from that of mRNA. Coupled with our results, different assumptions can be made. Since PD1 is expressed in TILS, and our samples had lower expression of TILs infiltration, this may explain the non-expression of PD1. Based on PD-L1 and TILs expression, Teng et al. classified the TME into four forms. PD-L1+/TILs+; PD-L1+/TILs-; PD-L1^-^/TILs+ and PD-L1^-^/TILs-. The last study indicated the implication of other regulators of the anti-tumor immune response (50).

The prognosis of TILs in UM is a matter of reflection from various studies and reviews. Indeed, their presence may predict the response to treatment, and is linked to a better outcome in several types of cancers. The tumor mutational burden was assumed to be reflected by the infiltration levels of TILs. Singh et al. established that TILs infiltration is related to worse prognosis in UM, suggesting that patients with decreased infiltration of TILs may benefit from the effectiveness of immunotherapy (21). It is not to forget that PD1 is not only expressed on T cells, but also on myeloid cells, thymocytes, and on B cells (38). Therefore, eliminating the fact that the absence of T cells may justify the absence of PD1 staining, the mRNA expression of PD1 was already high in patients with UM in TCGA cohort. This may be due to multiple biological processes and genetic mechanisms, such as post-transcriptional modifications, that may explain the lack of protein expression. The limited sample size included in our study might also have influenced this expression.

Furthermore, while seeking to elucidate PD1 and CTLA-4 survival analysis, we noticed that both high PD1 and CTLA-4 expression decreased patient survival. Although statistically significant, they did not show beneficial outcomes in clinical trials for primary or metastatic UM patients. In addition to the low response rates in retrospective data, as previously stated, treatment with anti-CTLA-4 antibodies, especially Ipilimumab and Tremelimumab, may have many immune-related adverse events and unwanted side effects. A meta-analysis of 81 fully reviewed articles reported that most immune-related adverse events of anti-CTLA-4 treatment occur as skin lesions (dermatitis, epidermal spongiosis, and Sweet’s syndrome), hormonal deficiencies, hepatitis, colitis, pancreatic abnormalities, neurologic complications, ocular diseases, visual disturbances, and severe immune complications (51).

To go more in depth in our study, T cells gene expression profile, their infiltration, and their association with VISTA expression were studied; since many studies have reported that UM microenvironment is infiltrated by CD3, CD4 and CD8 lymphocytes (52–55). According to TCGA database, strong infiltration of CD8+ T cells, CD4+ T cells and regulatory T cells (FoxP3) is noticed in the tumor microenvironment of UM patients expressing high levels of VISTA. Besides, regulatory cytokines like IL-10 and TGFB, play an important role in modulating the immune system, as IL-10 regulates T cell proliferation (56, 57). In line with our findings, they were found to be overly expressed in UM microenvironment, creating an immunosuppressive microenvironment. Suggesting that VISTA may play a compelling role in creating an immunosuppressive microenvironment as well as in the induction of regulatory T cells overexpression.

In order to study the functional role of CD8+ T cells infiltrating our microenvironment, we attempted to characterize their phenotype among UM patients. As reported by Sun et al., persistent carcinogenesis may lead to a T cells phenotype termed “exhausted”; the presence of strong infiltration of these exhausted CD8 + T cells induce the promotion of immune evasion in UM (58–61). Additionally, Chen and Mallmen identified EOMES ^+/-^, TBET ^+/-^ and PD1 ^med^ as a phenotype for recoverable and exhausted effector CD8+ T cells markers (62). These data are consistent with our findings that report high expression of EOMES, TBET, and PD1 in the presence of high VISTA. EOMES deletion was found to impact the exhaustion process, confirming that it is associated with the terminal differentiation of exhausted CD8+ T cells (63). Here we suggest the potential role of VISTA in T cell exhaustion in UM microenvironment; it may therefore be considered as a marker of exhaustion in UM. Surprisingly, patients with higher VISTA gene-level RNA-seq expression showed the worst survival, which was dramatically decreasing. However, lower VISTA mRNA expression was associated with better outcomes (P= 0,0003) (**Figure 7**). Comparatively, promising results were obtained when blocking VISTA in murine models of various tumors (16, 19). Taken together, we hypothesized that VISTA expression may play a pivotal role in the survival of patients with UM. This might explain the failure of anti-PD1 therapy and anti-CTLA-4 inefficiency in clinical trials of patients with UM. Our study suggests an alternative pathway by which malignant cells may escape the immune response.

Our findings show that VISTA may be a possible immune checkpoint in patients with malignant UM. Its blockade might be a better potential treatment among different immune checkpoint inhibitors that have previously failed in patients with UM. Evidently, other studies might be necessary to determine whether VISTA may be combined with other immune checkpoint inhibitors to achieve an optimal strategy leading to long-term survival of patients with UM.

## Data availability statement

The original contributions presented in the study are included in the article/supplementary material. Further inquiries can be directed to the corresponding author.

## Ethics statement

The studies involving humans were approved by Institutional Ethics Committee of Biomedical Research of Casablanca (N°04/21), Faculty of Medicine and Pharmacy, Casablanca. The studies were conducted in accordance with the local legislation and institutional requirements. The participants provided their written informed consent to participate in this study. Ethical approval was not required for the study involving animals in accordance with the local legislation and institutional requirements because animals were not used in this study.

## Author contributions

Conceptualization: NIIS and AB; Data curation: NIIS, FM, MK, and MEB; Formal analysis: NIIS; Funding acquisition: AL and AB; Investigation: FM, MEB, and AB; Methodology: NIIS, and AB; Project administration: MEB and AB; Resources: NIIS, FM, MK, and MEB; Software: NIIS and AB; Supervision: FM, MEB, and AB; Validation: FM, MEB, and AB; Writing original draft: NIIS; Writing review & editing: FM, MEB, and AB. All authors contributed to the article and approved the submitted version.
